# 2471. Comparison of Dialysis Infection Events in Outpatient End Stage Renal Facilities Pre- and During the COVID-19 Pandemic: Louisiana, 2018-2021

**DOI:** 10.1093/ofid/ofad500.2089

**Published:** 2023-11-27

**Authors:** Grace Lee, Erica Washington, Ashlyn Pinkins

**Affiliations:** Louisiana Office of Public Health, New Orleans, Louisiana; Louisiana Office of Public Health, New Orleans, Louisiana; Louisiana Office of Public Health, New Orleans, Louisiana

## Abstract

**Background:**

Bloodstream infections (BSIs) in patients with end-stage renal disease (ESRD) undergoing hemodialysis (HD) are associated with significant morbidity and mortality. While robust infection prevention and control (IPC) education has lowered BSI rates in the last decade, the COVID-19 pandemic posed significant challenges in terms of IPC practices. The aim of this review is to determine if COVID-19, and associated strains on IPC capacity, impacted dialysis events (DEs) in outpatient ESRDs in Louisiana.

**Methods:**

Dialysis events and associated patient-months data from the National Healthcare Safety Network (NHSN) Dialysis Event Surveillance Module from January 2018 to December 2021 were extracted to compare outcomes in patients receiving HD from free-standing ESRD facilities in Louisiana in the time preceding the COVID-19 pandemic (January 2018 to February 2020) and during the COVID-19 pandemic (March 2020 to December 2021). Incidence density rates (IDR) and relative risks (RR) were computed in the NHSN Statistics Calculator and openepi.com, respectively, for the retrospective cohort. Statistical significance was considered at the 95% confidence level.

**Results:**

From 2018 to 2021, 189 facilities reported 12,583 DEs to NHSN. The pre-COVID IDR (184.22/10,000 patient-months) was higher than the COVID IDR (173.85/10,000 patient-months; *p*< 0.001). ESRD patients with DEs were 23% more likely to be hospitalized pre-COVID in comparison to the COVID period (95% CI: 1.18-1.27), and were 4% more likely to die due to DE before COVID, though this risk was not statistically significant (95% CI: 0.92-1.17). Independent free-standing ESRD facilities had a lower rate of DE (15.03/10,000 patient-months) compared to corporate facilities (357.66/10,000 patient-months; *p*< 0.001).

**Conclusion:**

Despite disruptions in healthcare delivery and IPC practices during the COVID-19 pandemic, indicators of BSIs as reported to NHSN did not worsen during this period. This may be attributed to heightened awareness of vulnerabilities exposed to dialysis patients during the pandemic and increased IPC awareness despite constraints. Granularity with respect to data reviews is warranted due to PPE and staffing constraints over the study period to determine if risk differences were variable during the COVID period.

**Disclosures:**

**All Authors**: No reported disclosures

